# Assay of honey freshness by a novel optical technique

**DOI:** 10.1038/s41598-022-04920-w

**Published:** 2022-01-18

**Authors:** Alireza Mashhadi, Ali Bavali, Farzad Mokhtari

**Affiliations:** Department of Energy Engineering and Physics, Amirkabir University of Technology, Tehran, 15875-4413 Iran

**Keywords:** Fluorescence spectroscopy, Characterization and analytical techniques

## Abstract

Assay of Maillard reaction products (e.g. furosine) is a reputable method for determination of the honey freshness. In this report, novel optical technique is proposed for real-time measurement of the changes of furosine content in honey. The method is based on the unidirectional energy transfer between two peaks of the doubled-peak fluorescence spectrum as secondary inner filter effect (2nd-IFE) in a specific arrangement of the laser induced fluorescence (LIF) setup. Proper optical parameters are defined accordingly, and affirmed to be dependent on the content of furosine in honey. It is shown that the introduced parameters are not sensitive to the LIF intensity fluctuations induced by the ambient noises and particularly alter due to the 2nd-IFE. Furosine level of 8 honey types with different botanical origin were chemically determined before and after the 1 year storage, and compared with the values of the devised spectral parameters. Proofs conducted that the proposed technique can be utilized for evaluation of the honey freshness.

## Introduction

As one of the high nutritional value foods, honey is a complex matrix of several compounds including carbohydrates (76–85% depending of the water content), free amino acids (~ 1 g/kg), proteins (~ 5 g/kg), and other minor components e.g. enzymes, minerals, vitamins and pollen grains^1^. However, improper factory conditions such as prolonged storage or heating (in order to delay crystallization) lead to conversion of the carbohydrates into the unfavorable chemical agents. The most pronounced chemical is 5-hydroxymehyl-2-furaldehyde (HMF), which is formed during the progress of Maillard reactions and/or caramelization due to pasteurization, liquefaction or long-term storage at room temperature. In early steps of the Maillard reactions, Schiff’s bases are produced due to the reaction of reducing sugars with the amino group of the amino acids, peptides, or proteins^3^. Generation of the Schiff’s bases results in the formation of an Amadori compound, ɛ-N-Deoxylactulosyl-L-lysine, which can be partially converted via acid hydrolysis to a stable compound, “ɛ-N-2-furoylmethyl-L-lysine” known as furosine^2–4^. Furosine (C_12_H_18_N_2_O_4_) is an alpha amino acid in which the amino group is attached to the alpha carbon atom next to the carboxylate group. This intermediate product, does not necessarily require much heat to be generated and can be produced significantly over a long period of time at room temperature^5^. Therefore, besides HMF, furosine has been regarded as a sensitive indicator of the freshness of stored food products^5–10^. For instance, it has been shown that furosine and lactulose content are reliable indicators of the damage of ultra-high temperature (UHT) milk^4,7,11–13^.

There are also reports on the formation of the furosine in honey due to overheating or prolonged storage^2,14^. Using High Performance Liquid Chromatography (HPLC), Villamiel et al. measured an increase of the furosine content (~ 14.00 g/kg protein) in overheated honey samples (90 °C, 135 min)^2^. Cardenas-Ruiz et al. ^14^ utilized HPLC technique to reveal a remarkable raise in the furosine concentration of the heated honey. Based on similar results, assay of the furosine content as a precise indicator has been recommended to determine the quality of honey^15,16^.

Essentially, the most frequently employed techniques for quantification of the furosine content in honey are HPLC^14,16^ and capillary zone electrophoresis (CZE)^17^. However, those chemical-based techniques are time-consuming, costly and require preliminary chemical preparation of the sample^16,17^. Therefore, a demand exists for a simple, low cost, handheld and fast method for real-time monitoring of the furosine content during the heat treatment and/or storage of honey. In this regard, optical methods are well-adapted among which, fluorescence spectroscopy would be a proper choice due to its high sensitivity, accuracy, selectivity, simplicity, high speed and low cost^18–20^.

However, in a complex medium such as honey, fluorescence spectroscopy in its conventional form is not reliable due to the inner filter effects (IFEs), fluorescence quenching and multiple scattering of the photons^21^. Such screening events cause the relation between the measured fluorescence intensity and the fluorophore concentration to be aberrant of linearity^21,22^. IFEs may occur in two ways; (1) successive absorption of the exciting photons in medium such that a less intense light reaches the subsequent layer of the medium (primary IFE), and (2) partial reabsorption of the fluorescence emission in medium i. e. 2nd-IFE^23^. Besides IFEs, relatively larger particles, such as pollen grains and sugar crystals in natural honey, may aggravate the intensity distortions by providing scattering events.

Moreover, presence of the various fluorophores in honey and likelihood of the light scattering events (due to granularity), cause the detected LIF emission intensity to be significantly different from the original one. Though several analytical methods have been proposed to correct IFE noises, those corrections are not practicable in the case of the selective measurement in optically heterogeneous, multi-fluorophoric media^24,25^. Xu et al.^24^ demonstrated that photon scattering and absorption events have a distinct efficacy on IFE so that their contributions must be decomposed before making corrections. Accordingly, as will be discussed below, it is not possible to measure the amount of furosine in honey by conventional fluorescence spectroscopy.

Honey contains variety of fluorescent molecules with absorption/emission spectra covering ultraviolet to infrared spectral region^26^. The absorption spectra of some species significantly overlap with the emission spectra of the other one, so that the adsorbent species reabsorb the fluorescence emission of the emitting molecules. Based on a report by Lenhardt et al., spectral ranges of the excitation/emission of the major fluorescent components in honey can be classified as presented in Table 1. Most of the fluorescent species are phenolic compounds that contain one or more hydroxyl groups bonded directly to an aromatic hydrocarbon group. Depending on the structure of the phenolic compounds, their absorption and subsequent emission spectra cover different values in the range of 240–360 nm, in such a way that the simpler structures e. g. benzoic acid in group (I) absorb and emit light at shorter wavelengths and more complex structures e. g. Gallic acid in group (II) and Quercetin in group (III) at longer wavelengths^28^.

**Table 1:** Excitation/emission spectral regions of major fluorescent components in honey^26–28^.

Fluorophore	λ_exc._ (nm)	λ_em._ (nm)
Phenolic compounds* (I) e.g. Benzoic acid (C7H6O2)	240–265	370–495
Phenolic compounds (II) e.g. Gallic acid (C7H6O5)	280–320	390–470
Phenolic compounds (III) e.g. Quercetin (C15H10O7)	310–360	370–470
Aromatic amino acids	260–285	320–370
Maillard reaction products (mainly HMF and Furosine)	360–435	440–520
Riboflavin (also known as vitamin B_2_)	Visible range: 400–510	480–750

*Phenolic compounds are classified based on their chemical structure and corresponding absorption/emission peaks^28^.

**Figure 1:**
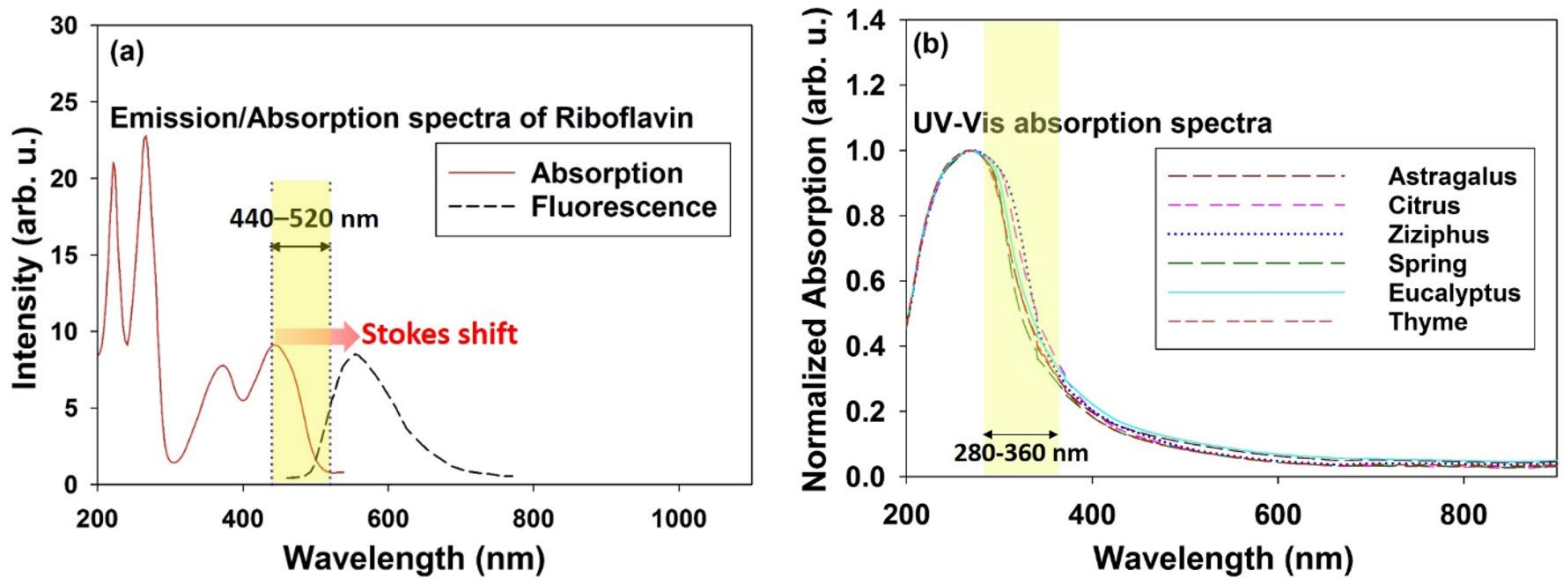
(**a**) Absorption/Emission spectra of the aqueous riboflavin; Fluorescence spectral range of the Maillard's products (440–520 nm) is highlighted. (**b**) UV–Vis spectra of some fresh honey types. Spectral range of 280–330 nm (highlighted by yellow colored) is related to the absorption by phenolic compounds.

According to Table 1, the fluorescence spectral range of the Maillard’s products (i. e. 440–520 nm) has a significant overlap with the absorption spectrum of riboflavin. Moreover, Fig. 1a demonstrates absorption/emission spectra of the aqueous riboflavin. It is clearly seen that the riboflavin can provide significant 2nd-IFE for the fluorescence emission of the Maillard products (e. g. Furosine). Therefore, furosine concentration cannot be accurately determined by direct excitation of the furosine and measuring the corresponding fluorescence intensity.

Figure 1b represents UV–Vis spectra of the different types of fresh honey samples. Highlighted area by yellow color (230–360 nm) which manifests the largest difference between the spectra, corresponds to absorption by phenolic compounds^26^.

Here, proper spectral parameters are introduced regarding the LIF spectroscopy of honey, that appoint a quantitative correlation between the reabsorption events (as 2nd-IFE) and the amount of furosine in honey, based on which the changes of furosine content in honey can be determined. It is shown that the proposed parameters particularly alters due to the reabsorption events and are not sensitive to the other screening factors and ambient noises.

## Methods and experiments

### Laser induced fluorescence spectroscopy

**Figure 2:**
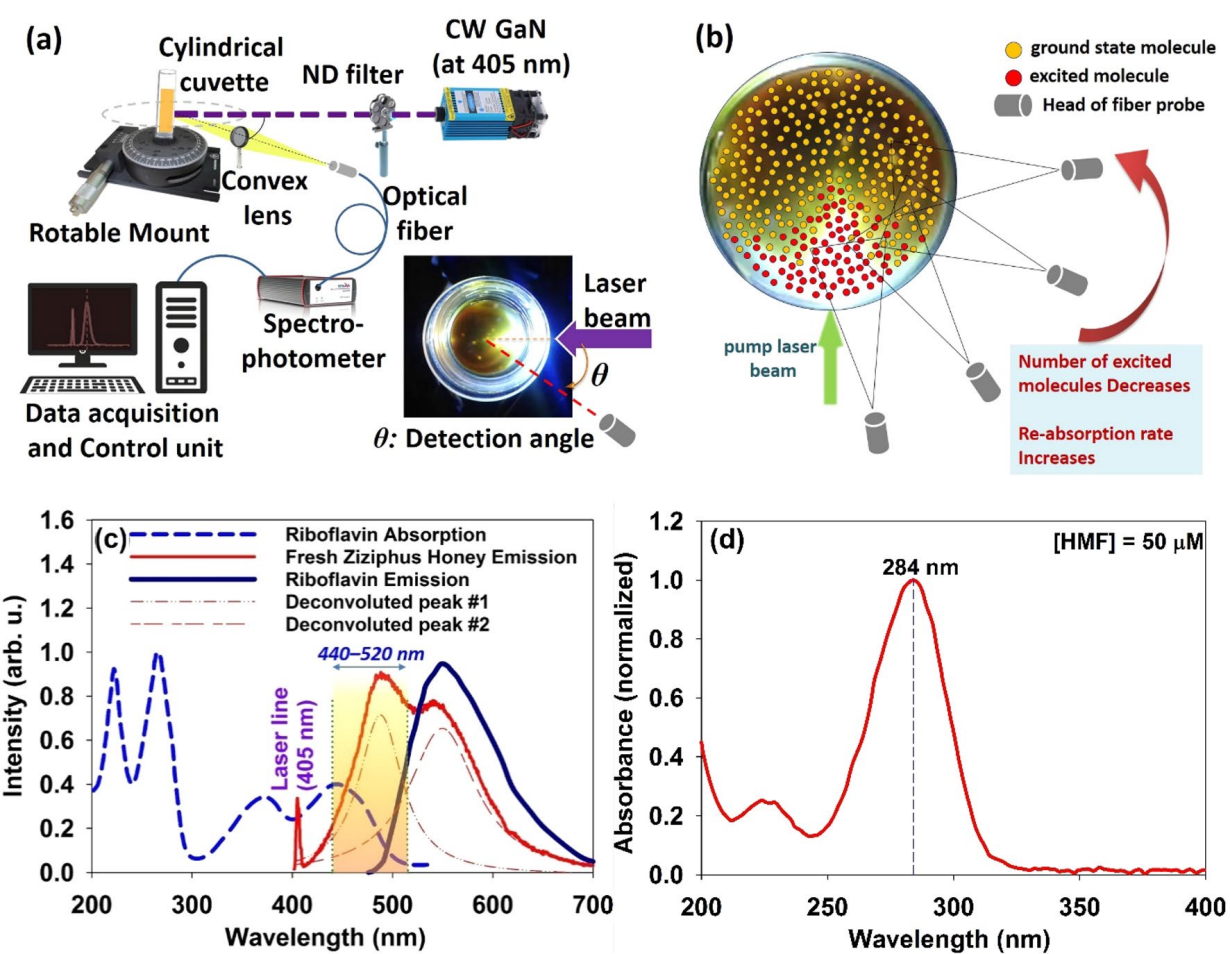
(**a**) Schematics of LIF measurement setup at cylindrical configuration. (Inset: definition of the detection angle on a top view of cylindrical glass cuvette). (**b**) Schematic distribution of the ground state and excited molecules in a typical active medium after illumination by a narrow beam of laser (from the top view of the cuvette). (**c**) Typical LIF spectrum of the fresh Ziziphus honey with excitation laser line (at 405 nm) recorded at detection angle θ = 60°; Two deconvoluted peaks are included. UV–Vis/fluorescence spectra of the aqueous riboflavin have been appended and absorption range of the Maillard's products (440–520 nm) is highlighted. (**d**) UV–Vis spectrum of the HMF (50 μM aqueous solution) with a significant maximum around 284 nm.

 Three sets of LIF experiments were carried out: (1) studying angular dependence of the LIF spectral features in order to characterization of the 2nd-IFE. (2) LIF spectroscopy of the fresh and stored samples of different honey types under a fixed LIF setup. (3) LIF spectroscopy of the fresh and heated samples of a typical honey type under a fixed LIF setup. Note that the LIF spectra were measured in experiments (2) and (3) at a certain detection angle. As demonstrated in Fig. 2a, all LIF measurements were performed at cylindrical configuration. Each sample was inserted into a cylindrical glass cuvette with 10 mm inner diameter and 1 mm thick to eliminate Fabry–Perot cavity effects. The cuvette was also slightly tilted to omit unwanted reflections of the laser light from the internal surface of the cell. To excite the honey, a continuous-wave (CW) GaN diode laser with 300 mW power at 405 nm wavelength and constant spot diameter of ~ 1 mm was exploited. Using proper ND filters, laser beam energies ranging 50–300 mW were examined quite below the degradation threshold of the honey ingredients. The optimal power of 200 mW was chosen to irradiate the samples to achieve measurable optical response while all being irradiated with the same power.

Laser wavelength was selected at 405 nm in accordance with the significant absorbance of the riboflavin and furosine molecules in which the absorption by sugars, phenolic compounds and aromatic amino acids is lowest (Table 1 and Fig. 1b). Laser beam was directed at the center of the cuvette surface to excite the fluorescent molecules, The emission was collected by a convex lens with 10 cm focal length and 1 cm diameter, and was detected using a UV–Visible-NIR spectrophotometer, Avantes AvaSpec-Mini 2048L, with spectral resolution of 0.1 nm. The SMA-905, UV600/660 fiber probe with NA = 0.22 (acceptance angle of 12.7°) that was mounted on a rotatable arm along the radial axis of the cell, was placed at a fixed distance (typically 1 cm) from the surface of the cell to detect the emission coming out of the medium at any desired angle respect to the direction of the laser beam.

Now it is required to mention the origin of the 2nd-IFE of the LIF in present setup. As depicted in Fig. 2c, typical LIF spectrum of the fresh Ziziphus honey excited at 405 nm wavelength (detected at angle θ = 30° respect to the laser line) comprises two main peaks: left peak (#1) at ~ 485 nm and the right peak (#2) at ~ 540 nm. UV–Vis/Fluorescence spectra of the riboflavin aqueous solution is also appended to assess the likelihood of the reabsorption events.

Based on data available in Table 1, left peak is mainly arises from the Maillard reaction products. Moreover, it is known that HMF molecules do not have significant absorption above 310 nm^29^ (see also Fig. 2d) while furosine significantly absorbs around the 405 nm^26^. Accordingly, pumping at 405 nm could not excite the HMF and the emission peak at 485 nm (left deconvoluted peak) mainly arises from the furosine content in accordance with the previous reports^26^. Moreover, as demonstrated in Fig. 1a, deconvoluted peak on the right is obviously related to riboflavin. Considering absorption region for Maillard products, it appears that LIF emission from excited furosine molecules (left peak) is significantly exposed to the reabsorption by the furosine (self-absorption) and riboflavin molecules in the ground state.

In angular measurements, LIF spectra were recorded at various detection angles respect to the laser beam direction (θ) to investigate the angular dependence of the spectral features. In the following, it is shown that the angular assessment of the LIF spectral features provides information regarding the inner filter of the furosine’s fluorescence emission by riboflavin. In Fig. 2b, top view of the spatial distribution of ground state and excited fluorophores in a cylindrical cuvette is depicted after illumination of the medium by a narrow beam of laser. In the present LIF setup where the medium is locally pumped by a narrow laser beam, exciting photons accumulate in front of the cuvette due to the scattering events inside the medium (notice that the honey is a turbid medium due to granularity). As a result, the excited volume would be concentrated in the vicinity of the laser beam and around the entry area. On the other hand, though fluorescence is emitted in all directions, but the reabsorption mainly occurs by non-excited absorbent molecules outside the excitation volume. Consequently, much stronger reabsorption is expected to occur at larger detection angles where more non-excited molecules are congested within the detector’s field of view. Another important point is that at certain detection angles (e.g. 60°) at which the received photons experience significant 2nd-IFE, if the concentration of the fluorophores increases, the rate of reabsorption and corresponding spectral shift will increase. This has already been explained in detail regarding the LIF spectroscopy of dye solutions that confront significant self-absorption (e.g. Rhodamine 6G and Coumarin 7 with small Stokes shift^30^).

Considering Beer–Lambert law regarding the reduction of the fluorescence intensity due to the reabsorption by a given type of absorbent molecules, the initial fluorescence intensity at wavelength λ, $F_{0} (\lambda )$, reduces to

1 $$F(\lambda ,dl) = F_{0} (\lambda )\exp \left( { - \sigma_{reabs} \left( \lambda \right)N_{Absorbent}^{GS} (l)dl} \right)$$

after passing distance *dl* in medium, where σ_reabs._(λ) and $N_{Absorbent}^{GS} (l)$ ascertain the reabsorption cross section at wavelength λ and density of the non-excited absorbent molecules respectively. Based on Eq. (1), σ_reabs._(λ) and $N_{Absorbent}^{GS} (l)$ modify the reabsorption rate. Overlapping area between the absorption and emission spectra determines σ_reabs._(λ). In fact, the larger the crossover area leading to enhance the reabsorption rate^30^. As the reabsorption events occur, preferential absorption at the shorter wavelengths gives rise to non-intrinsic red shift of the fluorescence peak toward the longer wavelengths (see spectral overlap area between riboflavin’s absorption and fresh honey’s emission spectra in Fig. 2c). On the other hand, at the larger detection angles, fluorescence photons diffuse through the congestion of more non-excited molecules (larger $N_{Absorbent}^{GS} (l)$) that are away from the pathway of the exciting laser beam (the medium is locally pumped by a narrow laser beam).

In the case of honey, though laser beam at 405 nm excites both furosine and riboflavin molecules, only furosine emission is subjected to significant reabsorption. In fact, negligible crossover area of the riboflavin’s absorption/emission spectra indicates that riboflavin is not subjected to significant self-absorption. However, according to Fig. 2c, the overlapping of the furosine emission with the riboflavin absorption spectrum portends the likelihood of the strong reabsorption of furosine emission by ground state riboflavin molecules outside the excited volume. This unidirectional energy transfer from furosine to riboflavin, which takes place in the present setup due to nonhomogeneous pumping. Notice that this event leads to the subsequent re-emission of the fluorescence by riboflavin molecules. This process is a basis for defining an appropriate optical parameter for quantitative assessment of the honey freshness.

### Spectra analysis

 For accurate characterization of the LIF spectra, convoluted peaks were recovered by OriginPro2019b package (Copyright©1991–2019, OriginLab, Corporation) that uses nonlinear Lorentzian curve fit on the basis of Levenberg–Marquardt iteration algorithm. Statistical assay of the retrieved data was performed using the standard paired t-test based on Shapiro–Wilk normality test in SigmaPlot v.14 package.

### Sampling

 73 unprocessed honey samples including 8 types of native honey with different botanical origin: Astragalus, Citrus, Eucalyptus, Multi-Floral, Spring, Thyme, Zirfon and Ziziphus were collected directly from beekeepers in Iran, on September 2019. All samples were stored at 25 ± 2 °C before commence the experiments. A number of fresh honey samples were stored at room temperature for a period of 1 year. A unique feature of the introduced method for measuring the amount of furosine is that the honey sample does not need any preparation, dilution or chemical process. In experiments regarding the effect of temperature rise on the fluorescence spectrum of the honey, a hot plate of Karazma model KH450 made in Iran, with a temperature range between 50 and 300 °C was used in order to heat the typical citrus honey sample. Honey sample was heated at 80 °C for 60 min. As the sample cooled, the LIF measurements were carried out at room temperature.

### UV–Vis spectroscopy

 To measure the absorption spectra, each honey sample was first diluted with distilled water at 40 °C and then poured into 1 cm × 1 cm × 4 cm quartz cuvette following cooling to room temperature. The UV–Vis absorption spectrometer model V-550, made by JASCO (United States), available in the Food Research Laboratory of the faculty of chemical engineering, Amirkabir University of technology was employed. This spectrometer has a resolution of less than 1.0 nm, and a 2000 Multichannel detector. The data acquired by spectrometer were analyzed by a computer equipped with Spectra Manager software. HMF (5-Hydroxymethyl-2-furaldehyde with 99% purity) purchased from Sigma-Aldrich and the standard solution (50 μM) was prepared with deionized water for determination of UV–Vis absorption spectrum of the HMF.

### Chemical specifications

 In order to verification of the results, standard methods were utilized to measure the content of furosine and HMF. For determination of the furosine content, HPLC analysis was accomplished based on the method of Delgado et al. ^14,31^. Measurement of hydroxymethylfurfural (HMF) in fresh and heated citrus honey was performed based on the White method^32^.

## Results

The experimental results are represented in the following order. First, reabsorption of the furosine emission by riboflavin molecules (as 2nd-IFE) has been investigated through an angular measurement of the spectra. Subsequently, LIF emission of the stored honey has been characterized based on the specific optical parameters that are defined with regard to the reabsorption process. Finally, results of the LIF spectroscopy regarding the heated citrus honey as well as several samples of two honey types provided from different geographical areas but with the same botanical origin have been assessed. Chemical measurements have been accomplished to verify the results.

### Angular dependence of the 2nd-IFE in fresh honey

**Figure 3:**
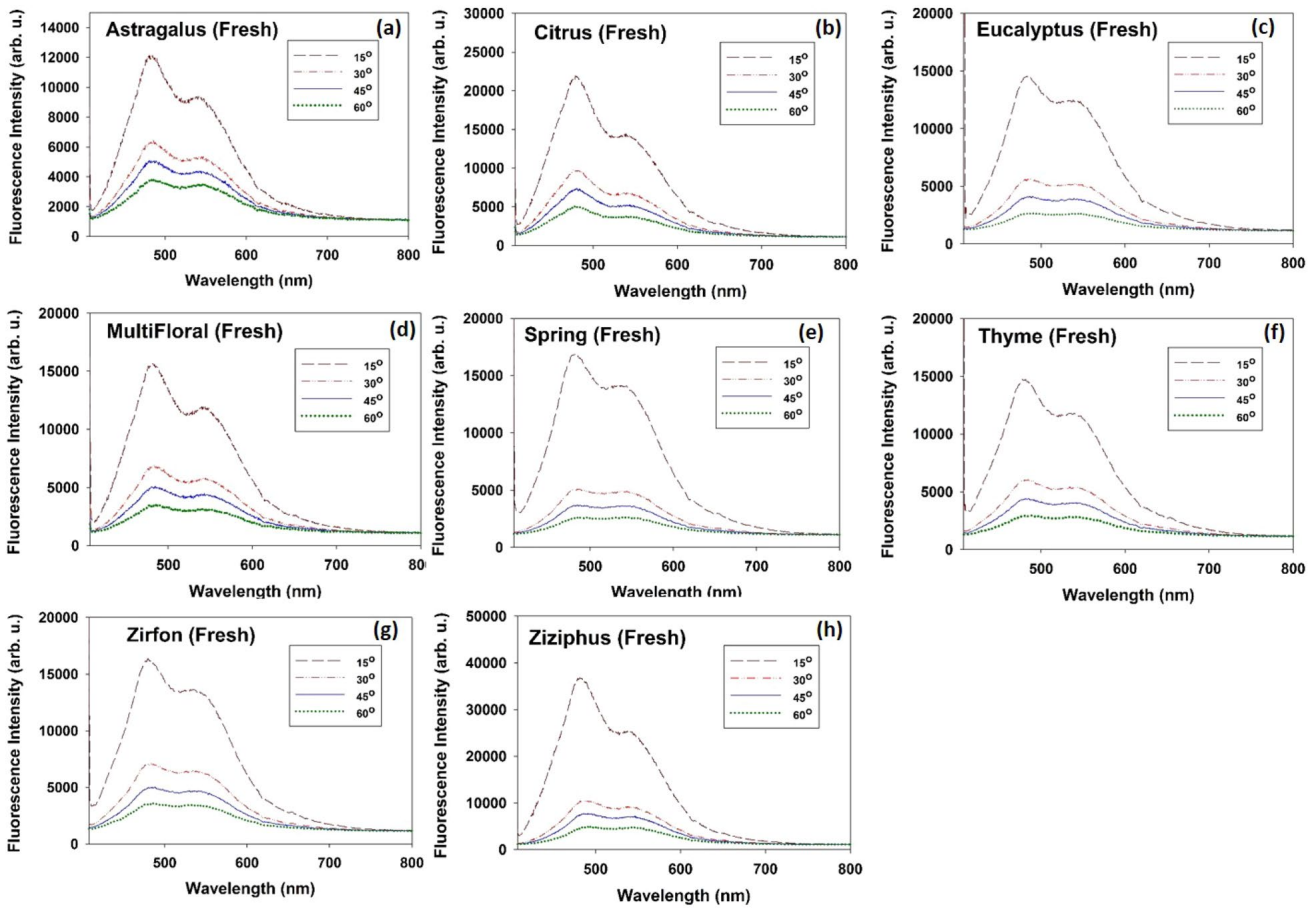
LIF spectra due to excitation of fresh honey, (**a**) Astragalus, (**b**) Citrus, (**c**) Eucalyptus, (**d)** Multi-Floral, (**e**) Spring, (**f**) Thyme, (**g**) Zirfon and (**h**) Ziziphus, by 405 nm diode laser; recorded at different angles 15°, 30°, 45° and 60° .

 Fig. 3a–h depict typical LIF spectra of fresh honey samples with different botanical origin (Astragalus, Citrus, Eucalyptus, Multi-Floral, Spring, Thyme, Zirfon and Ziziphus) recorded at different detection angles 15°, 30°, 45° and 60°.

**Figure 4:**
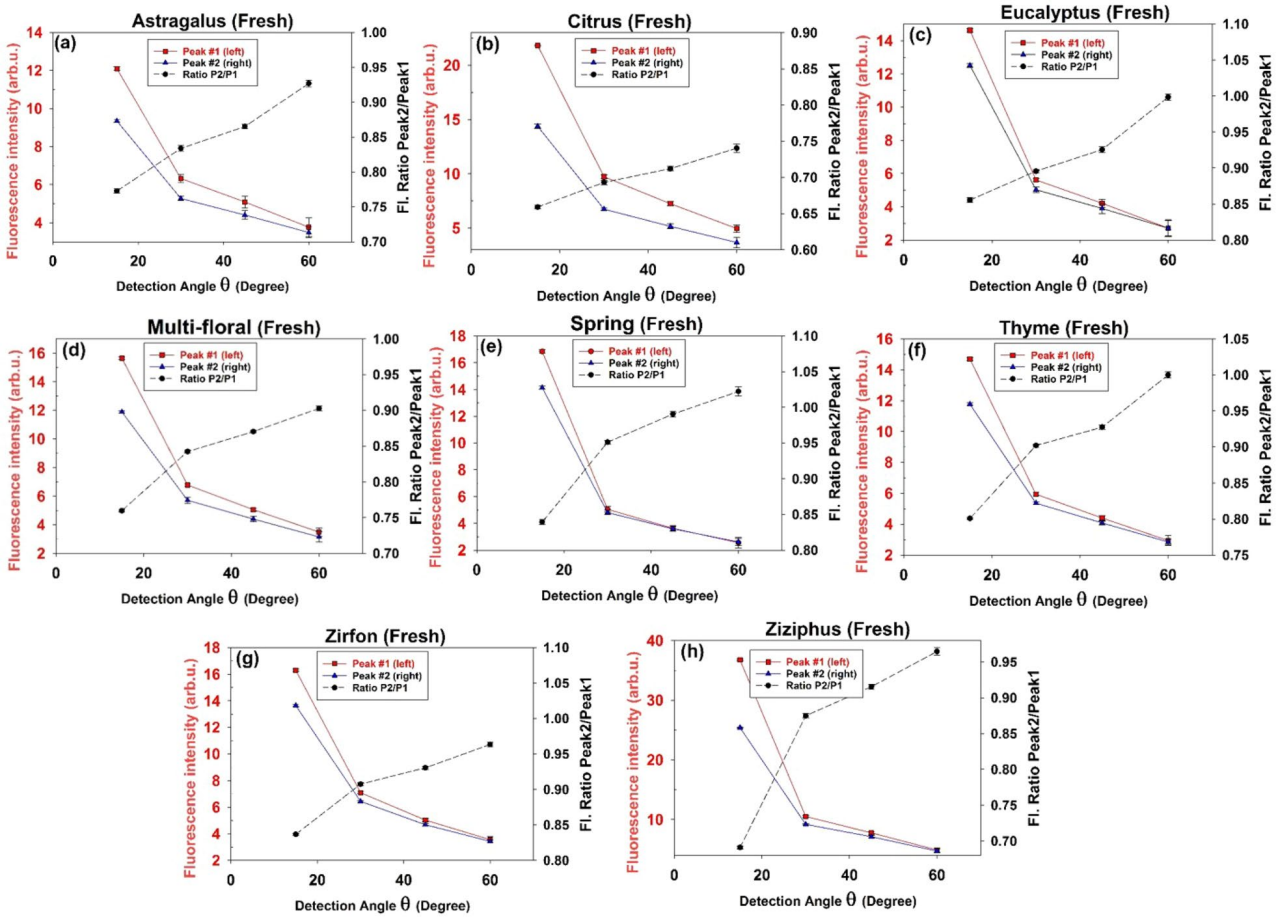
Intensities of the left (filled square, red color) and right (filled triangle, blue color) peaks of the doubled-peak LIF spectra of fresh honey samples and corresponding Intensity ratio I_Peak#2_/I_Peak#1_ (filled circle, black color) versus the detection angle (θ = 15°, 30°, 45° and 60°).

**Figure 5:**
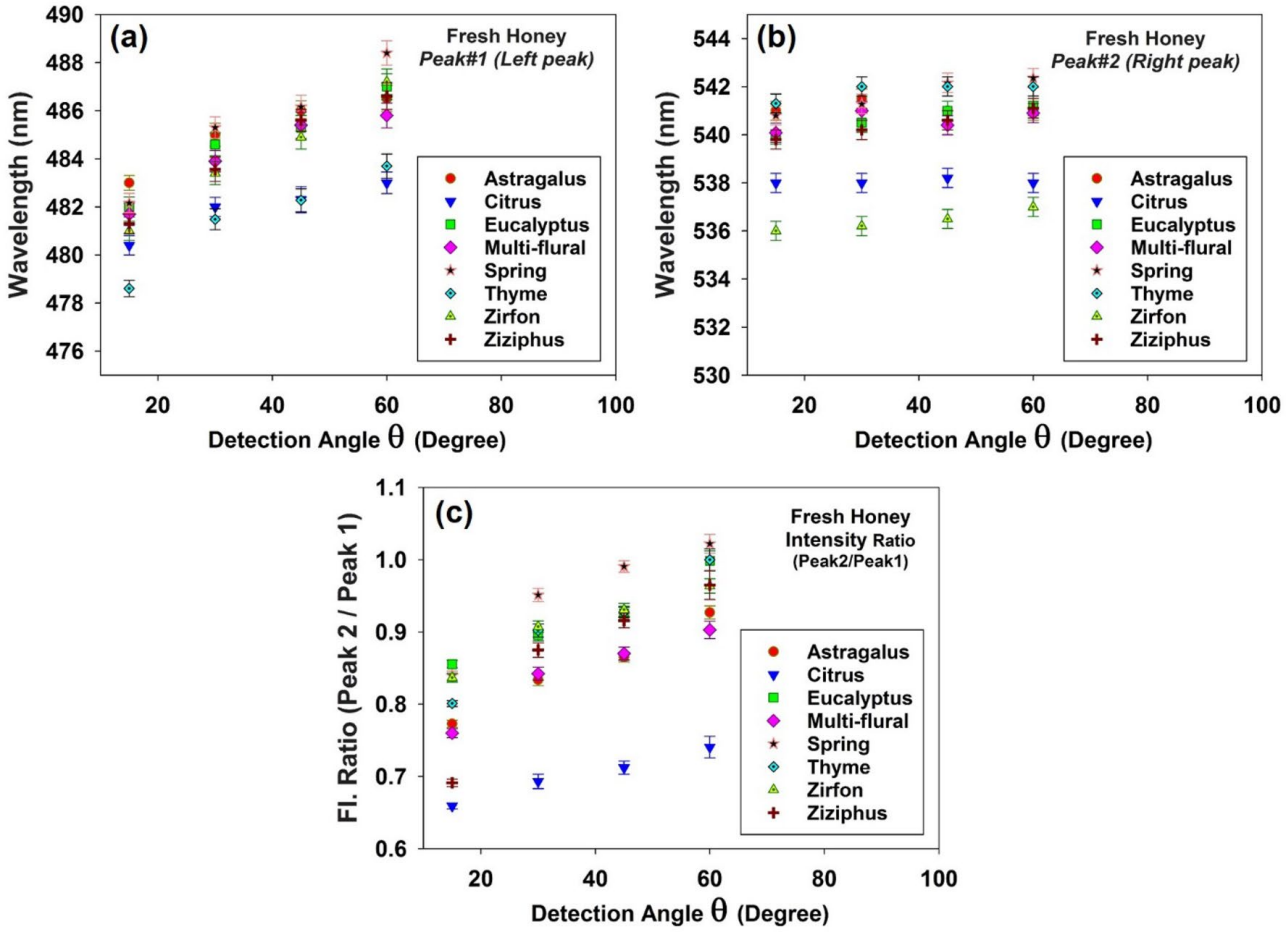
Wavelength of the (**a**) left and (**b**) right peaks of the LIF spectra versus detection angle (θ = 15°, 30°, 45° and 60°) regarding the fresh honey samples. The left peak experiences larger spectral red shift than the peak on the right due to stronger reabsorption events (as 2nd-IFE). (**c**) Intensity ratio of the peaks (I_Right peak_/I_Left peak_) for 8 fresh honey types versus the detection angle (θ = 15°, 30°, 45° and 60°). In all honey types the left peak experiences much larger increase than the peak on the right.

Plotting the intensity and corresponding wavelength in terms of the detection angle, one can realize angular dependence in both peak intensity and wavelength (Figs. 4 and 5). In general, the fluorescence intensity decreases versus the observation angle. As shown in Fig. 5a,b by increasing the detection angle spectral red shift occurs for both peaks. Notice that the left peak (peak#1) experiences larger red shift due to its wider overlap area with the riboflavin absorption spectrum (see Fig. 2c). Moreover, riboflavin molecules reabsorb the furosine emission and re-emit fluorescence with different quantum distribution which does not overlap with the furosine’s absorption spectrum. As a consequence, loss for furosine leads to the gain for riboflavin. According to Fig. 5c, as the detection angle increases, intensities of two peaks decrease unequally such that the ratio I_Peak#2_/I_Peak#1_ increases (in all the honey types the decrease in intensity of the left peak is much greater). Such an angular dependence could be explained based on the anisotropic reabsorption of the fluorescence emission by non-excited riboflavin and furosine molecules outside the excitation volume.

In summary, both riboflavin and furosine molecules in the pathway of the laser beam are most likely excited while those located out of the pumped volume remain in the ground state; therefore the larger observation angle corresponds to the more reabsorption events for the left peak of the fluorescence emission (furosine emission), leading to the more intensity reduction for this fluorescence peak and subsequent larger red shift. Moreover, unidirectional energy transfer from furosine to riboflavin leads to loss for furosine’s emission and gain for riboflavin’s emission. As a result, I_Peak#2_/I_Peak#1_ increases not only because of the reabsorption of left peak, but also due to the enhancement of the right peak. Therefore, both parameters I_Peak#2_/I_Peak#1_ and λ_peak#1_ should be considered for characterization of the detected fluorescence spectra.

### LIF spectra of the stored and fresh honey samples

**Figure 6:**
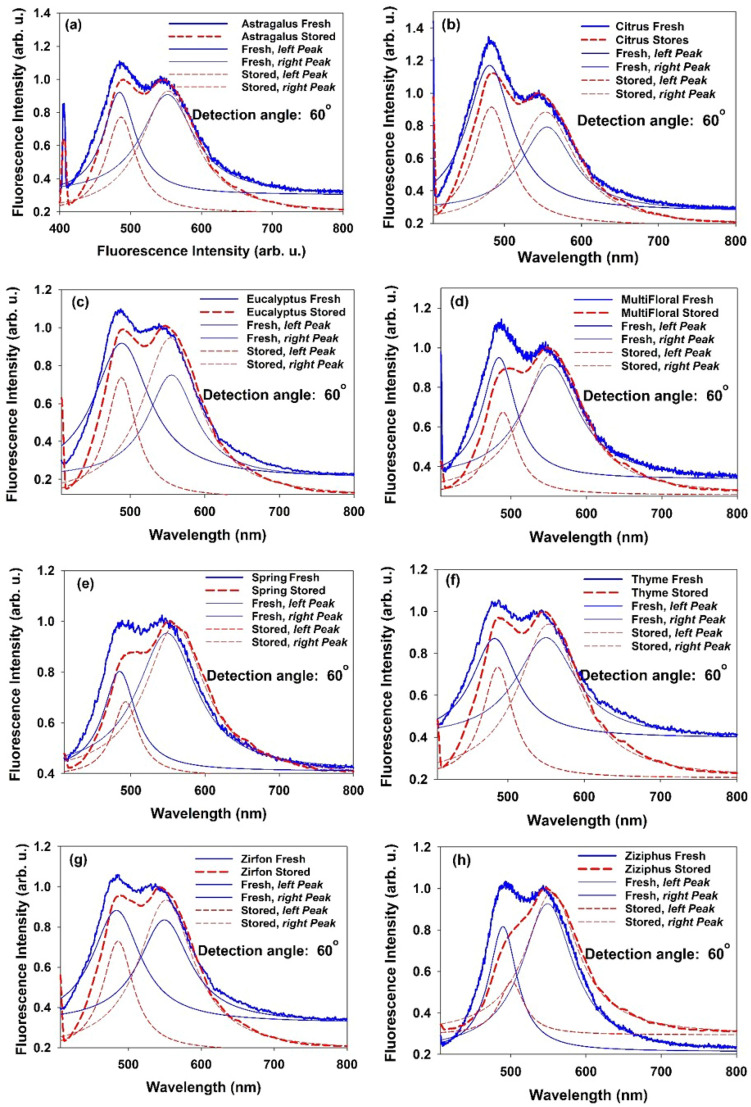
LIF spectra and corresponding deconvoluted peaks of the fresh and stored samples of 8 honey types. Detection angle of θ = 60° was chosen to measure the significant alteration in intensity and the related spectral shift.

**Figure 7:**
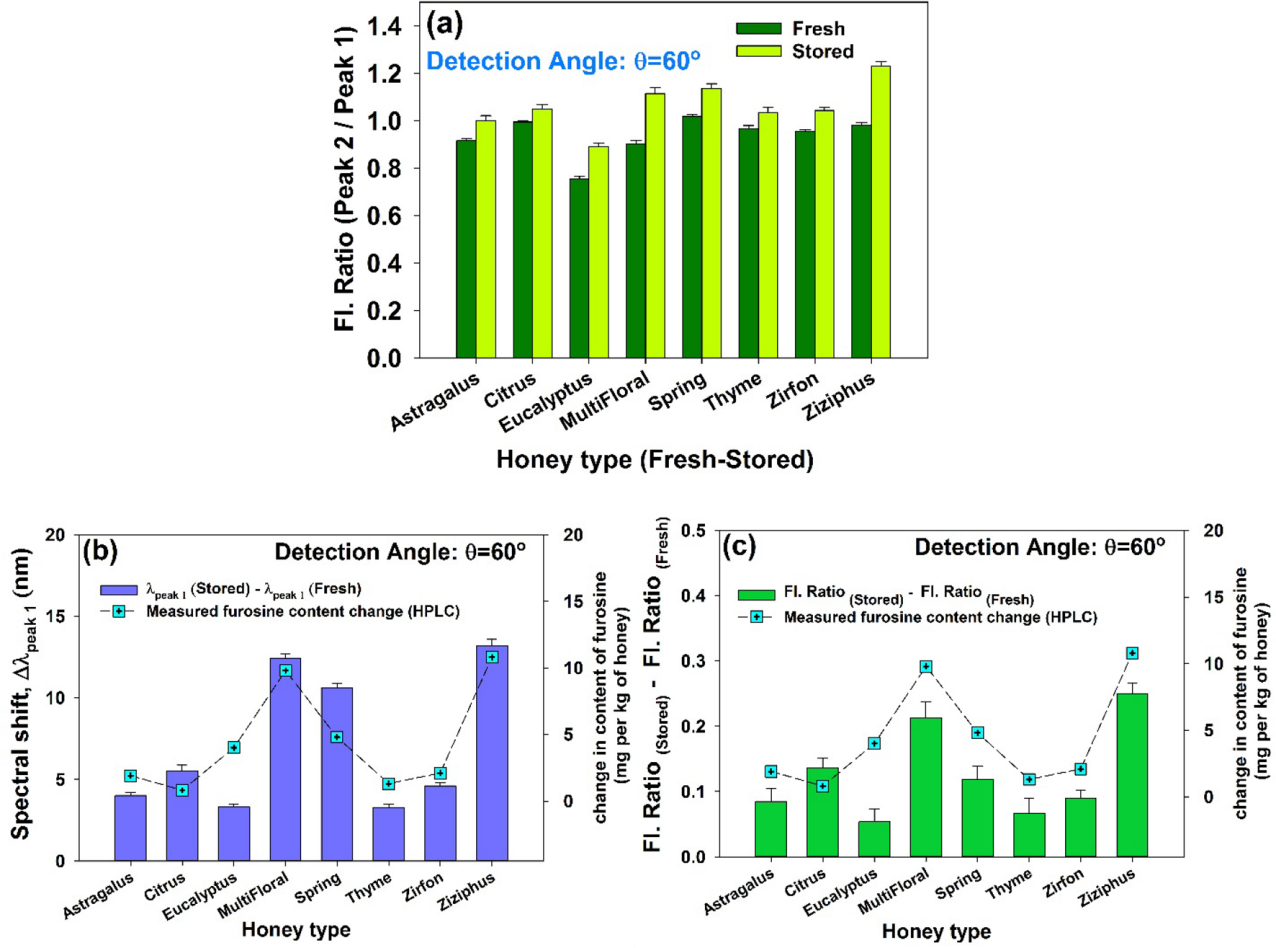
(**a**) Right peak-to-left peak intensity ratio (I_peak#2_/I_peak#1_) of the LIF spectra due to excitation of fresh and stored honey samples of 8 type. Detection angle of 60° was chosen to measure the significant alteration in intensity. (**b**) Spectral shift (red shift) of the left peak (peak 1) and (**c**) Change in the intensity ratio, [I_peak#2_/I_peak#1_ (Stored)—I_peak#2_/I_peak#1_ (Fresh)], due to excitation of fresh and stored honey samples of 8 type at detection angles θ = 60°. Measured furosine content is denoted for comparison. (Samples were stored at 25 ± 2 °C for 1 year).

 In order to establish a quantitative relation between the 2nd-IFE of LIF and the content of furosine, Fresh and stored samples of different honey types were subjected to LIF spectroscopy. According to the results of angular assessment, detection angle of 60° was chosen in order to encounter the significant change of the intensity and corresponding spectral shift. Figure 6a–h depict LIF spectra and the corresponding deconvoluted peaks of the fresh and stored honey samples. For each honey type, right peaks of the fresh and stored samples were normalized in order to better comparison with the left peaks. It is observed that for all honey types, LIF intensity ratio (I_peak#2_/I_peak#1_) of the stored honey is greater than that of fresh one (see Fig. 7a). In addition, for all types of honey, LIF peak of the stored sample is red-shifted respect to the fresh one (see Fig. 7b,c).

In order to elucidate these result, the amount of furosine in fresh and stored samples were determined by direct chemical measurements. Table 2 provides the furosine content in fresh and stored (1 year) samples of different honey types which has been measured utilizing HPLC technique.

**Table 2:** Furosine content in fresh and stored honey samples measured by HPLC technique.

Honey type	Furosine content (mg in 1 kg honey)	
	Fresh	Stored (1 year)
Astragalus	13.8	15.7
Citrus	11.6	12.3
Eucalyptus	18.4	22.4
Multi-Floral	21.3	31.1
Spring	22.5	27.3
Thyme	35.6	36.9
Zirfon	17.3	19.4
Ziziphus	31.7	42.5

As explained in the methods section, the observed non-intrinsic spectral red shift is due to the reabsorption events as 2nd-IFE. In other words, spectral red shift of the left peak is correlated to asymmetric change in the intensity of the fluorescence spectrum (increase in I_peak#2_/I_peak#1_). Figure 7b,c ascertains such a correlation to be used for the assessment of the honey freshness. In Fig. 7b,c two parameters i.e. spectral shift and intensity ratio (I_peak#2_/I_peak#1_) are compared, respectively, with the measured changes in the amount of furosine (based on data presented in Table 2) for different types of honey. It follows that the change in furosine content in honey, whether it is fresh or stored, strongly depends on the honey type. This result is consistent with the reports based on direct measurement of furosine in different honey types utilizing chemical techniques^2,15^.

**Figure 8:**
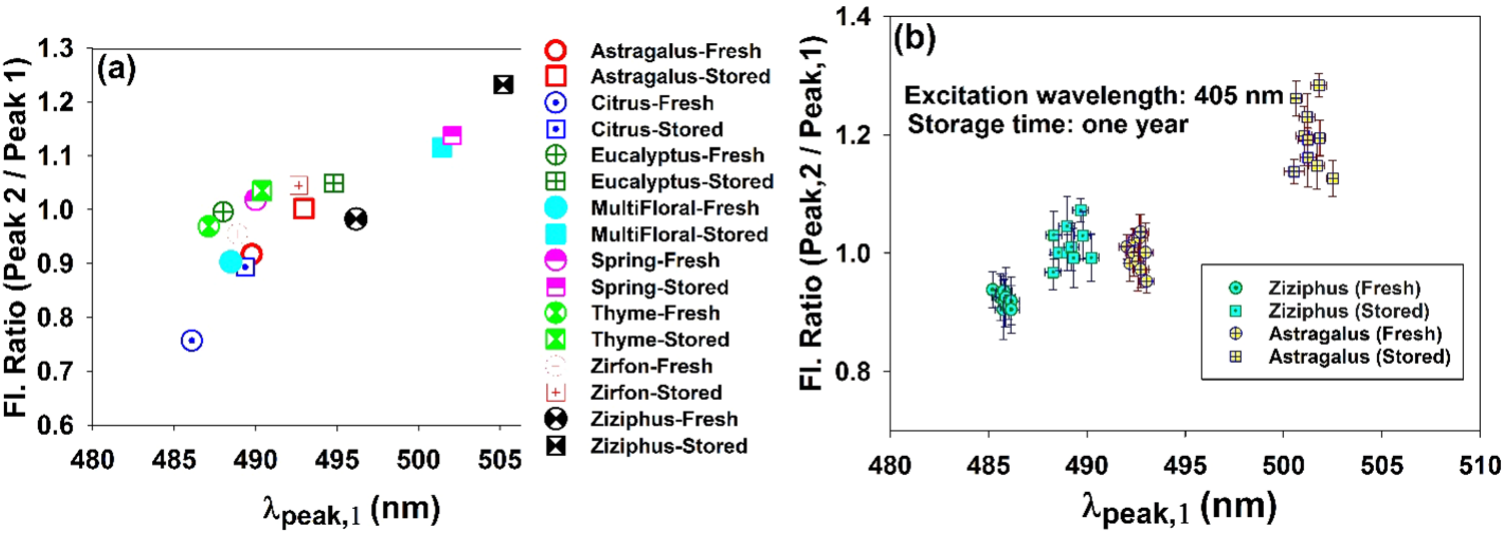
(**a**) LIF intensity ratio (I_peak#2_/I_peak#1_) versus the wavelength of the peak 1 (left peak) for both fresh and stored samples of different honey types. (**b**) Distribution of the honey samples with different geographical areas but with the same botanical origin (Ziziphus and Astragalus), in the diagram of ((I_peak#2_/I_peak#1_ vs. λ_peak,1_)).

Figure 8a depicts intensity ratio (I_peak#2_/I_peak#1_) versus the wavelength at the left peak (peak#1) of fresh and stored samples of different honey types. Different types of honey (both fresh and stored samples) are located in different parts of the phase space.

For each type of honey, distance between the fresh and stored points is proportional to the amount of furosine produced after 1 year. To assess the accuracy of the method, samples of two honey types provided from several beekeepers in different geographical areas (but with the same botanical origin) were examined. Figure 8b demonstrates the distribution of honey samples taken from different geographical areas but with the same botanical origin, in I_peak#2_/I_peak#1_ versus λ_peak,1_ diagram. As can be seen, statistical error of calculating Ipeak#2/Ipeak#1 (averaging the measured data) is higher than that of λpeak,1 because emission intensity is affected by several factors (reabsorption, light scattering and ambient noises), while emission wavelength is mainly determined by reabsorption events^30^.

### LIF spectroscopy of the heated and fresh honey sample

**Figure 9:**
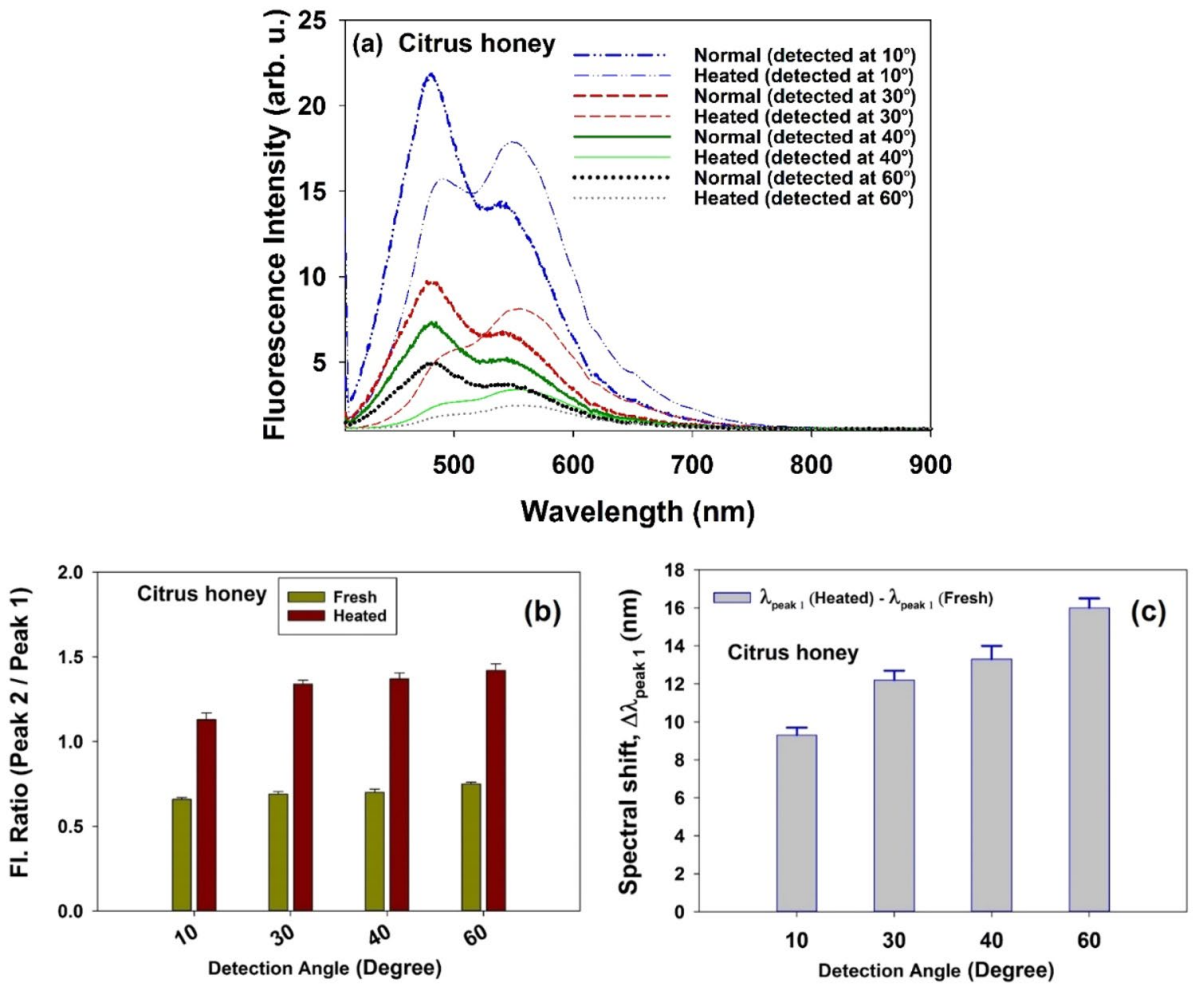
(**a**) LIF spectra due to excitation of fresh and heated citrus honey samples at detection angles 15°, 30°, 45° and 60° (**b**) I_peak#2_/I_peak#1_ of LIF spectra due to excitation of fresh and heated citrus honey samples and (c) corresponding spectral shift (red shift) at various detection angles θ = 15°, 30°, 45° and 60° (samples were heated at 80 °C for 60 min).

 Finally, fluorescence features of the heated citrus honey was examined. Figure 9a represents LIF spectra due to excitation of the fresh and heated (at 80 °C) citrus honey samples at detection angles 15°, 30°, 45° and 60°. In general, LIF emission of the heated sample is less intense. Similar to results of the storage experiments, at each detection angle, a comparison of two samples reveals that right peak-to-left peak intensity ratio (I_peak#2_/I_peak#1_) of the heated sample is greater and encounters a notable spectral red shift relative to the fresh one. Moreover, consideration of the angular dependence reveals that the 2nd-IFE enhances as the angle increases (Fig. 9b,c), which is in harmony with the results of the angular assessment.

It is worth noting that both spectral red shift and the difference in the intensity ratio (I_peak#2_/I_peak#1_) between fresh and heated citrus samples are much greater respect to that for fresh and stored samples.

Measured amounts of furosine/HMF were 11.5/20.8 (mg in kg of honey) for fresh and 28.3/175.0 (mg in kg of honey) for heated honeys, respectively. Drastic increase in HMF content with regard to the severe heating condition is admissible^33^. Also, the content of furosine is more than twice its initial amount, which is in accordance with the significant increase in the value of I_peak#2_/I_peak#1_ and corresponding spectral shift.

## Discussion

**Figure 10:**
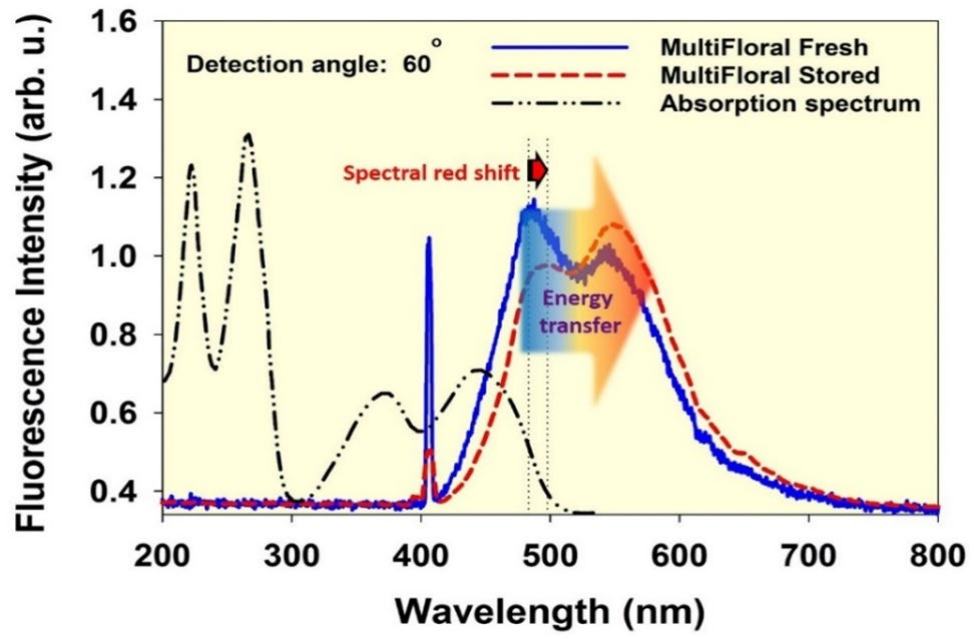
Typical LIF spectra of the fresh and stored multifloral honey samples excited by laser at 405 nm and recorded at angle θ = 60°. UV–Vis spectrum of riboflavin aqueous solution have been appended.

Honey as a high nutritional value product is an intricate blend of several components e. g. carbohydrates, organic acids, aminoacids, pollen, lactones, minerals, enzymes and vitamins. Quality of honey is determined based on various parameters among which the content of furosine indicates the extent of the storage time or heat treatments. Furosine is produced in honey due to acid hydrolysis of the fructosyl-lysine that is generated following the Maillard reactions over time. Therefore, it can be a reliable indicator for assessment of the freshness of honey. Under the illumination of a given honey sample in a cylindrical cuvette by a narrow beam of CW-diode laser at 405 nm, Maillard reaction products (especially furosine) and riboflavin molecules are excited (utilized wavelength does not excite phenolic compounds, aromatic amino acids and HMF to fluorescent emission). Those excited molecules in pumped area (mainly riboflavin and furosine) emit fluorescence photons in all directions. The emitted photons that have passed through the excited region are probably reabsorbed by ground state riboflavin and/or furosine molecules outside the excitation volume. Following Eq. (1), one can write relations regarding the wavelength dependent loss terms for fluorescence emission of the riboflavin and furosine molecules after traveling an average distance $\overline{d}$ before leaving the medium as follows:

2 $$loss_{Fur.} (\lambda_{Fur.}^{fluor.} ,\overline{d}) \propto \exp \left( { - \int\limits_{0}^{{\overline{d}}} {\left[ {\sigma_{abs.}^{Rib.} \left( {\lambda_{Fur.}^{fluor.} } \right)N_{Rib.}^{GS} (l) + \sigma_{abs.}^{Fur.} \left( {\lambda_{Fur.}^{fluor.} } \right)N_{Fur}^{GS} (l)} \right]dl} } \right)$$

3 $$loss_{Rib.} (\lambda_{Rib.}^{fluor.} ,\overline{d}) \propto \exp \left( { - \int\limits_{0}^{{\overline{d}}} {\left[ {\sigma_{abs.}^{Rib.} \left( {\lambda_{Rib.}^{fluor.} } \right)N_{Rib.}^{GS} (l)} \right]dl} } \right)$$

where $\overline{d}$ is the mean travelling length of the emitted photons at wavelength $\lambda^{fluor.}$, $\sigma_{abs.}$ is molecular absorption cross-section, $N^{GS}$ is the number density of ground state molecules as a function of position and the indices *Fur.* and *Rib.* ascertain the riboflavin or furosine species respectively. Based on Eqs. (2) and (3), in addition to the absorption cross-section, the abundance of ground state molecules along the way of photons is also crucial parameter in determining the amount of loss. Notice that $\sigma_{abs.}^{Fur.} \left( {\lambda_{Rib.}^{fluor.} } \right)$ is negligible according to Fig. 2c and data presented in Table 1, therefore its contribution in Eq. (2) was eliminated. Moreover, it should be accentuated that loss for furosine emission ultimately leads to an increase in the fluorescence intensity of riboflavin. The process is graphically represented in Fig. 10.

Reabsorption of the fluorescence emission mainly occurs within the spectral range of the furosine’s fluorescence (see Fig. 2c). As the amount of riboflavin in honey is almost constant over time, rise of the furosine content during the storage time enhances the rate of reabsorption (as 2nd-IFE). As a result, significant reduction of the furosine emission intensity and corresponding spectral red shift occurs in addition to increase of the riboflavin fluorescence intensity due to enhancement of the unidirectional radiative energy transfer from excited furosine to ground state riboflavin. In fact, over time, two significant changes occur in the double-peaked fluorescence spectrum of the stored honey, as measured by the specified LIF spectroscopy arrangement presented in this report: (1) spectral red shift of the left peak (furosine contribution) (2) increasing the intensity ratio of the right peak (riboflavin contribution) to the left peak (furosine contribution). First event is a result of the reabsorption (as 2nd-IFE) of furosine emission outside the excited volume, which enhances by increasing the furosine concentration. The second event arises from the unequal changeover of the intensities of two peaks such that the intensity of the left peak decreases (due to 2nd-IFE) while intensity of the right peak enhances (due to unidirectional radiative energy transfer from excited furosine to ground state riboflavin).

Furosine level of native honey samples including 8 types with different botanical origin i. e. Astragalus, Citrus, Eucalyptus, Thyme, Zirfon, Ziziphus (6 unifloral honeys) as well as Multi-Floral and Spring (2 multifloral honeys) were determined before and after the 1 year storage using standard HPLC analysis (see Methods section). Furosine formation over time (ranging 0.7–10.8 mg per kg of honey) is shown to be correlated with characteristic spectral changeover of doubled-peak fluorescence spectra i.e. red shift of the left peak (4.0–13.2 nm) and increase of the intensity ratio, i. e. Δ(I_Right peak_/I_Left peak_) ~ 0.05–0.24. It is remarkable to compare the measured values of furosine content with those provided by other reports. Table 3 represents the reported values of the furosine content generated due to either heating or long storage of the honey^2,14,16^. In general, alteration of the furosine content during the storage or heating varies depending on the honey type and different protein content.

**Table 3:** Comparison of the measured furosine content with those provided by other reports.

Authors	Honey type	Method	Furosine formation mechanism		Generated content (mgkg^−1^ honey)	
Villamiel et al.^2^	Rosemary	Reversed phase liquid chromatography	Heat treatment at 50 °C for:	45 min	11.7	
				90 min	14.8	
				135 min	19.5	
Cárdenas-Ruiz et al.^14^	Multi-floral	Ion-pair reversed phase liquid chromatography	Heat treatment at 70 °C for:	5 min	0.2	
				15 min	7.1	
				45 min	22.3	
Morales et al.^16^	Various types (not-specified)	Ion-pair reversed phase liquid chromatography	Heat treatment at 50 °C for:	5 h	1.6	
				10 h	4.7	
				24 h	9.9	
				29 h	7.5	
				34 h	3.2	
			Storage at room temperature	2 years	Type 1	3.7
					Type 2	3.1
					Type 3	15.5
This report	Citrus	HPLC/LIF	Heat treatment at 80 °C for 60 min			16.8
	Astragalus	HPLC/LIF	Storage at room temperature		1 year	1.9
	Citrus					0.7
	Eucalyptus					4
	Multi-Floral					9.8
	Spring					4.8
	Thyme					1.3
	Zirfon					2.1
	Ziziphus					10.8

Statistical analysis was performed for a total of 73 samples used in the experiments. Utilizing standard paired t-test based on Shapiro–Wilk normality test in SigmaPlot v.14 package it was obtained that at the 99% confidence level, intensity ratio (I_peak#2_/I_peak#1_) increases after 1 year storage and the wavelength of the left peak of doubled-peak fluorescence encounters significant red shift in stored honey sample respect to the fresh one. In addition, scrutiny of the amount of furosine generated in different honey types (which has been chemically measured) ascertain that the amount of furosine produced in each type of honey corresponds to the rate of changes of two spectral parameters at the 73% confidence level.

Plot of LIF intensity ratio (I_peak#2_/I_peak#1_) versus the wavelength of the left peak (λ_peak,1_) enables various honey types to be distributed according to the furosine content. It is well known that the different honey types contain different protein content. Moreover, the protein content of honey is directly related to its furosine content^2,3,15^. As a result, it is reasonable to assess the introduced optical parameters in order to make proper set of principle components and recast the honey types along the principal components axes. However, in order to accomplish the analysis, for each honey type a large number of the samples from different geographical areas must be examined in order to perform the various steps of the principle component analysis (PCA) i. e. standardization, computation of the covariance matrix and assessment of the feature vector with sufficient accuracy. This idea could be the subject of a future study on the discrimination of the different honey types based on the assessment of furosine and HMF content.

Assay of the data obtained from honey samples with different geographical areas but with the same botanical origin (i.e. Ziziphus and Astragalus) reveals that under the same conditions, the amount of furosine produced in a given type of unifloral honey does not depend significantly on geographical origin, so that those samples are localized in a limited area in the diagram of I_peak#2_/I_peak#1_ versus λ_peak,1_.

## Limitations

The present research was intended to be an empirical survey of randomly sampled unifloral and multifloral honeys, to ascertain a reliable optical technique for assessment of honey’s freshness.

The main limitation of the proposed method is that despite being simple, fast, real-time and low cost, the measurement is performed indirectly. As a result, a large number of data is required for preparing an accurate calibration curve. Since the amount of furosine and HMF formation are not necessarily the same in different honey types (due to the different content of sugars, proline and acidity), based on the present data set, no accurate quantitative relation can be made between the amount of furosine and the values of two specified spectral quantities. In fact, it is necessary to prepare and examine a larger number of unifloral and multifloral honey samples of different botanical origin from several geographical areas.

Another limitation arises from limited time interval (1 year). In fact, for each type of honey, the amount of furosine as well as the spectral quantities must be measured at regular intervals during the storage time. Time resolved measurements by chemical methods reveal that the amount of furosine in honey changes nonlinearly over time. Therefore, in addition to increasing the number of data, it is necessary to measure the optical parameters in several time intervals.

Finally, it is also necessary to determine the relationship between the heating rate and the changes in the spectral quantities and to compare them with the results of the long-term storage experiments.

## Data Availability

The datasets obtained by experiments and/or analyzed during the current research are available from the corresponding author on reasonable request.
